# Molecular, Systemic, and Physiological Adaptations to High-Intensity Interval Training in Flatwater Kayak Athletes

**DOI:** 10.3390/sports13120451

**Published:** 2025-12-12

**Authors:** Apostolos Papandreou, Georgios Tzanis, Athanasios Moustogiannis, Evangelos Zevolis, Elias Zacharogiannis, Maria Maridaki, Serafim Nanas, Michael Koutsilieris, Anastassios Philippou

**Affiliations:** 1 Department of Physiology, Medical School, National and Kapodistrian University of Athens, 11527 Athens, Greece; papandreouapostolis2020@gmail.com (A.P.); amoustog@med.uoa.gr (A.M.); ezevolis@med.uoa.gr (E.Z.); mkoutsil@med.uoa.gr (M.K.); 2 School of Physical Education and Sport Science, National and Kapodistrian University of Athens, 17237 Athens, Greece; elzach@phed.uoa.gr (E.Z.); mmarida@phed.uoa.gr (M.M.); 3 First Critical Care Medicine Department, “Evangelismos” Hospital, National and Kapodistrian University of Athens, 10679 Athens, Greece; giorgtz@yahoo.gr (G.T.); sernanas@gmail.com (S.N.)

**Keywords:** high intensity interval training, systemic adaptations, hormonal responses, physiological adaptations, flatwater kayak

## Abstract

Purpose: High-intensity interval training (HIIT) has emerged as a time-efficient alternative to traditional endurance training. This study investigated the molecular, systemic, and physiological adaptations induced by an 8-week HIIT program in national-level flatwater kayak athletes. Methods: Six trained male kayakers completed an 8-week HIIT intervention. Skeletal muscle biopsies and venous blood samples were collected before and after training to assess markers related to endocrine function, growth and remodeling, angiogenesis, and inflammation. Physiological and performance measures were evaluated using a maximal oxygen uptake (VO_2_max) test and kayak ergometer trials. Results: HIIT elicited significant improvements across molecular, systemic, and performance parameters. Post-training analyses showed increased expression of IGF-1R, MMP-4, MMP-9, and TNF-α (*p* < 0.05), along with elevated serum testosterone concentrations (*p* < 0.05). Notable performance gains were observed in paddling speed at the second ventilatory threshold (PSVT2; *p* < 0.05) and in 1000 m (*p* < 0.01) and 200 m (*p* < 0.001) time-trial performances. Conclusions: An 8-week HIIT program effectively enhanced molecular signaling, systemic adaptation, and sport-specific performance in elite flatwater kayak athletes. The concurrent upregulation of anabolic, remodeling, and inflammatory pathways suggests that HIIT facilitates coordinated muscular and systemic adaptations beneficial for kayak performance.

## 1. Introduction

High-intensity interval training (HIIT) consists of repeated short bouts of exercise, which are characterized by high intensity and intervals, and can be completed in a short time, though having the benefits of continuous endurance exercise training [1,2,3,4,5,6,7,8,9,10]. Indeed, HIIT has become popular due to its time effectiveness compared to traditional, more time-consuming forms of endurance training (ET) [11,12,13]. In upper-body-dominant sports such as kayaking, HIIT has been shown to induce significant improvements in muscular strength, power, and endurance within the shoulder, back, and arm muscles—key contributors to stroke performance [14,15]. These adaptations correspond closely with the sport’s biomechanical demands, where repetitive, forceful paddle strokes depend on strong upper-body musculature, coordinated core stability, and sustained power output [16,17,18]. By improving anaerobic capacity and neuromuscular efficiency, HIIT can enhance stroke force, paddling velocity, and overall racing performance, offering a time-efficient and targeted training strategy for kayakers [14,17,18].

HIIT is widely used by kayak athletes across training phases to enhance both aerobic and anaerobic performance. In sprint kayaking, HIIT is especially relevant for optimizing upper-body adaptations and exploring the physiological differences between arm-dominant and leg-dominant exercise modalities [14,15].

Although kayaking elicits relatively high peak oxygen uptake (VO_2_peak) values, these values are typically lower than those observed in sports dominated by lower-body work, such as cycling, rowing, or running [19,20]. For example, Billat et al. [21] reported that kayak ergometer power output at VO_2_peak represents only 57% of that achieved by cyclists. Similarly, Tesch et al. [22] found that oxygen uptake during 500 m and 1000 m kayak races corresponds to approximately 77% and 87% of VO_2_peak measured during leg exercise, respectively. Hahn et al. [23] also observed that kayak ergometer VO_2_peak values reach about 89% of those recorded on combined arm/leg ergometers, underscoring the limitations of upper-body oxygen delivery and utilization in kayaking [24,25,26].

Most studies, so far, have selectively focused on either physiological, or systemic or molecular responses to HIIT, and there is less information regarding an overall profiling of HIIT-induced adaptations, concurrently investigated in the same athletes [10,11]. Thus, it remains a challenge to characterize the simultaneous local and systemic responses of various muscle-associated proteins and hormones after HIIT, in the context of their potential interactions in regulating HIIT-induced adaptations. The purpose of this study was, therefore, to examine in parallel physiological and performance variables as well as key molecular and systemic factors in skeletal muscle biopsy and blood samples before and after an 8-week HIIT protocol in high-class kayakists. Flatwater kayak performance is determined by the interplay of biomechanical, neuromuscular, and physiological factors that optimize propulsion efficiency and energy expenditure. Biomechanically, stroke technique—including paddle trajectory, stroke length, and force application—is crucial for maximizing forward propulsion and minimizing energy losses due to lateral or vertical motion [14,15]. Neuromuscular coordination between the trunk, shoulder girdle, and lower limbs ensures efficient transfer of force from the paddle to the kayak, while rapid force generation and precise muscle activation maintain rhythm and power throughout race phases [14,15,18,19]. Physiologically, elite kayakers require high aerobic capacity to sustain submaximal workloads, substantial anaerobic power for high-intensity bursts, and strong lactate-buffering capacity to preserve stroke efficiency under fatigue [14,15]. Specifically, the anabolic responses of growth hormone (GH), its primary downstream mediator insulin-like growth factor 1 (IGF-1), and testosterone (Testo), which play a critical role in the formation, maintenance, and regeneration of skeletal muscle, were measured along with cortisol (Cort) and thyroid hormones, thyroid-stimulating hormone (TSH), and free tetraiodothyronine (free thyroxine; fT4) following the HIIT protocol. Thyroid hormones influence factors that control energy balance and can modulate metabolic rate in the mitochondria of skeletal muscle [27]. Thyroid hormones were included as exploratory variables to assess potential endocrine adaptations to HIIT. Although their responsiveness to short-term, high-intensity interventions is less well characterized than traditional metabolic markers, thyroid hormones regulate basal metabolic rate, substrate utilization, and energy expenditure. Their responses to HIIT are expected to provide insight into possible endocrine contributions to exercise-induced adaptations and recovery in sprint kayak athletes.

In addition, various potential components of the molecular signature of HIIT adaptations [13] were also examined in this study, i.e., the expression of growth factors belonging to the IGF-1 bioregulation system (IGF1Ea, IGF-1Eb and IGF-1Ec, IGF1-R, IGFBP-3), Peroxisome Proliferator Receptor-γ (PPAR-γ), tissue remodeling factors such as urokinase type plasminogen activator (UPA) and its receptor (UPA-R), transforming growth factor-β (TGF-β), matrix metalloproteinases (MMP)-4 and MMP-9, angiogenic factors such as vascular endothelial growth factor-A (VEGF-A), angiopoietin-2 (ANG-2), and angiopoietin-like protein-4 (ANGPTL-4), and hypoxia inducible factor 1a (HIF-1a), and inflammation-related factors such as interleukin (IL)-6, IL-8, and tumor necrosis factor-a (TNF-a) [28].

We expected that a two-month HIIT intervention in sprint kayak athletes would induce anabolic and angiogenic adaptations, reflected by increases in markers of muscle hypertrophy, growth factors, and capillary density. Concurrently, we hypothesized a reduction in inflammatory markers, indicating improved recovery and attenuation of exercise-induced systemic inflammation. By distinguishing these expected directions—enhancement of anabolic and angiogenic processes versus suppression of pro-inflammatory signaling—the study aimed to elucidate biological pathways through which HIIT promotes performance improvements in upper-body-dominant endurance sports like sprint kayaking.

## 2. Materials and Methods

### 2.1. Ethical Approval

Written informed consent was provided by all the volunteers to participate in this study, while for subjects under 18 years of age, both their own and parental written consent were obtained. The informed consent(s) was approved by the Ethics Committee of the National and Kapodistrian University of Athens (3422/Δ5 protocol code, 12 January 2022).

### 2.2. Participants

A public announcement was issued through the official website of the Hellenic Federation and disseminated via email to all sports clubs involved in sprint kayaking development in Greece. However, only a few athletes had the opportunity for a six-week period of absence from training (detraining) and competition (see below). A total of eight athletes initially fulfilled the inclusion criteria and enrolled in the study, of whom six completed it. Specifically, six healthy male national-level flatwater kayak athletes volunteered to participate in the study (age: 18 ± 4 years; body mass: 70.75 ± 7.13 kg; height: 176.33 ± 4 cm). Inclusion criteria were (1) adults aged 17–30 years, (2) national-level Greek male kayak sprinters with ≥4 years training experience, (3) able to provide informed consent, and (4) able to communicate in Greek. Exclusion criteria included (1) chronic health conditions requiring ongoing pharmacological treatment, (2) current participation in another intervention study, or (3) inability to complete study procedures. Before starting the training period, a six-week detraining phase was implemented for all participants. During this period, they engaged in restorative, submaximal-intensity activities (e.g., walking, jogging, or swimming) three times per week, with one rest day between sessions [29].

### 2.3. Experimental Design

**Figure 1:**
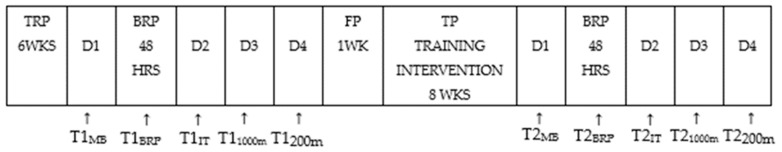
Schematic illustration of the experimental design of the study. Before entering the training program (TP), which lasted 8 WKS (see text for details), participants had completed a 6-WK transition recovery period (TRP). After the muscle biopsy (MB) collections (pre training-T1MB and post training-T2MB), a 48 HRS recovery period (BRP) took place. Participants were familiarized with the testing protocols and the kayak ergometer within a familiarization period (FP) of 1WK. The pre-training (T1) and the post-training phase (T2) consisted of 3 tests; the incremental test performed on D2 (T1IT), the 1000 m performance test on D3 (T11000 m), and the 200 m performance test performed on D4 (T1 200 m). After the 8-week training period, the post-training muscle biopsy was taken 48 HRS after the last exercise session, to detect the training effect and not the acute responses to HIIT exercise. On each muscle biopsy day and before the biopsy procedure, blood sample was also withdrawn.

The flatwater kayakers performed a HIIT training program for an 8-week training period, at a frequency of 3 times per week (24 sessions in total) with 24h off between the exercise sessions. Before starting the training period of this study, a six-week detraining period was applied to all participants [29] (Figure 1). An incremental maximal VO_2_max test was performed before (T1; Pre) and after (T2; Post) the intervention program using a kayak ergometer simulator (Stroke2max, Upington, South Africa), to measure the intensities of the training program as well as the potential effects of the HIIT protocol on selected physiological variables to be determined [30] (Figure 1). No strenuous exhausting exercise was undertaken 24 h before reporting to the laboratory for the trials testing. The same warm-up procedures and protocol for each test were repeated on subsequent occasions. All experimental procedure testing sessions were performed at the same time of the day (12.00–16.00) and under similar environmental conditions (20–21 °C and at 50–55% humidity) [30].

### 2.4. HIIT Protocol

In this study, the exercise training protocol lasted for 8 weeks: The HIIT protocol was 8 × 30 s paddling at 120% VO_2_max with 60 s passive recovery between the 8 repetitions. The training schedule was 3 training sessions per week with a day off. The training sessions for HIIT were on Monday, Wednesday, and Friday between 12.00 and 16.00, while Tuesday, Thursday, and Saturday were the days off. Training volume and intensity were carefully controlled and quantified by experienced canoeing coaches during each training session throughout exercise training protocol.

### 2.5. Somatometric Measurements

Participants’ body mass and height were measured using standard techniques and their body mass index (BMI) was then calculated. Body fat was assessed by skinfold thickness measurements using the Harpenden Skinfold Caliper (Batty International, London, UK) [31], and the body fat percentage (%) was calculated using the appropriate equation for men [32]. All skinfolds’ measurements were performed by the same technician using the same skinfold caliper.

### 2.6. Cardiorespiratory Fitness Assessment

The incremental VO_2_maxtest took place before (T1) and after (T2) the 8-week exercise training intervention (Figure 1). After a 5-min warm-up at ~70% HRmax, kayak athletes participated in an incremental paddling test to exhaustion on the kayak simulator [33,34]. Drag factor was stable at 70% of the flywheel air resistance scale (70–100%) of the ergometer (Stroke2max), and the first stage was set at a speed of 8 km·h^−1^, and the speed increments were set at 1 km·h^−1^ every 2 min. Each kayak athlete was allowed to freely adjust his stroke rate as needed to reach the target speed. Heart rate (HR) was monitored using standard telemetry (S610i; Polar Electro Oy, Kempele, Finland) and recorded every 5 s. Total time of the incremental test was 10–14 min [35]. Paddlers were encouraged to give their maximal effort and complete as many stages as possible [35]. Termination criteria included volitional fatigue or failure to maintain target speed. Secondary VO_2_max criteria were respiratory exchange ratio (RER) ≥ 1.10, HR within 10 beats·min^−1^ of predicted maximum, or HR ≥ 90–95% HRmax [36]. Gas analysis was conducted throughout the test using Cosmed equipment (CPET, Milan, Italy) according to the Douglas bag method. The test concluded when a participant voluntarily stopped paddling, or he was unable to maintain the target speed, while second degree criteria for VO_2_max and the speed at VO_2_max were further used, as described elsewhere [34].

### 2.7. Physiological Variables

Participants completed an incremental test (T1) to exhaustion on the kayak-type ergometer to determine maximum oxygen uptake (VO_2_max), peak blood lactate [La^+2^]peak, paddling speed at VO_2_max (PSVO_2_max), heart rate at VO_2_max (HRpeak), paddling economy speed (PES), which was the speed at 75% of maximum oxygen consumption, and paddling speed at the anaerobic ventilatory threshold VT2 (PSVT2) [37]. Exercise volume and intensity of the training program were quantified based on the incremental test preceded (T1), as previously described [15,35,36]. Capillary whole blood samples were taken from each kayaker’s fingertip, 3 min after the end of the incremental test to determine peak lactate concentration [La^+2^]peak (LP20 mini-photometer; Dr. Lange, Montpellier, France) according to the D-max method [38].

### 2.8. Blood Sampling and Serum Measurements

On each muscle biopsy day and before the biopsy procedure (Figure 1), blood samples were withdrawn from an antecubital vein of each participant at the same time of the day for all subjects (between 15.30 and 16.30). After 10 min in a supine position, 10 mL of blood sample were drawn and allowed to clot at room temperature for 30 min, and serum was collected after centrifugation at 4000 rpm for 10 min at 4 °C, stored frozen in 0.5 mL aliquots at −80 °C, and only thawed once for analysis.

Serum total IGF-1 was determined by a standard sandwich enzyme-linked immunosorbent assay (ELISA) protocol using a commercially available kit (Assay Designs, Ann Arbor, MI, USA) according to the manufacturer’s instructions. Similarly, serum GH, cortisol, total testosterone, thyroid stimulating hormone (TSH), and free tetraiodothyronine (fT4) concentrations were also determined by standard sandwich ELISA protocols using commercially available kits (GH, cortisol and testosterone: Enzo life Sciences, New York, NY, USA; TSH and fT4: MP Biomedicals, Santa Ana, CA, USA). The color formation was measured by a microplate reader (Versamax, Molecular Devices, San Jose, CA, USA) at 450 nm and calculations were carried out using SoftMax Pro 6.0 software (Molecular Devices, CA, USA). All samples were run simultaneously, analyzed in duplicate, and the results were averaged [24].

### 2.9. Muscle Biopsies and Tissue Processing

Skeletal muscle biopsies were taken from the middle deltoid under local anesthesia (2% lidocaine; AstraZeneca, London, UK) using a 5 mm Bergström needle. Biopsies were performed (between 15:30 and 16:30) 48 h before (T1) and 48 h after (T2) the final HIIT session. Participants followed a standardized light meal consumed three hours prior (60% carbohydrate, 25% protein, 15% fat). The second sample was taken ~2 cm from the first site to minimize interference. Samples (~70–100 mg) were snap-frozen in liquid nitrogen and stored at −80 °C for RNA analysis [27].

### 2.10. RNA Extraction and RT-PCR

Total RNA was extracted from 30–40 mg of muscle tissue and reverse transcription (RT) and semiquantitative real-time polymerase chain reaction (RT-PCR) were performed as previously described [27] to identify differences between mRNA expression before and after the 8-week training program. The primer set sequences used for the specific detection of each gene of interest are given in Table 1. Specifically, primers were designed against components of the IGF-1 bioregulation system (IGF1Ea, IGF-1Eb and IGF-1Ec, IGF1-R, IGFBP-3), PPAR-γ, TGF-β, uPA and its receptor uPA-R, MMP-4 and MMP-9, VEGF-A, ANG-2, ANGPTL-4, and HIF-1a, TNF-a, IL-6, and IL-8. Glyceraldehyde 3-phosphate dehydrogenase (GAPDH) was applied as housekeeping gene (internal standard).

### 2.11. Statistical Analysis

Data were first assessed for normality using the Shapiro–Wilk test. When the assumption of normality was satisfied, paired Student’s *t*-tests were conducted; otherwise, the Wilcoxon signed-rank test was used to evaluate pre-post differences in molecular, systemic, physiological, and performance variables. Effect sizes for parametric comparisons were calculated using Cohen’s d and interpreted according to established thresholds: negligible (<0.20), small (0.20–0.49), medium (0.50–0.79), and large (≥0.80). To complement numerical effect sizes, directional qualitative descriptors were applied to indicate both magnitude and direction of change (e.g., negligible, small–medium, no change, small, large). These descriptors were included in data tables where applicable. Ninety-five percent confidence intervals (95% CIs) were calculated when appropriate. Statistical significance was accepted at *p* < 0.05. No adjustment for multiple comparisons was performed due to the focused, hypothesis-driven nature of the analyses. All statistical procedures were conducted using GraphPad Prism version 2025 (GraphPad Software, San Diego, CA, USA).

## 3. Results

### 3.1. Somatometric Characteristics

Somatometric features of the kayak athletes are shown in Table 2. No significant pre-post training differences were observed in height, body mass, lean body mass (LBM), and BMI. Interestingly, significant differences were observed in the body fat percentage (%) between the values measured before and after the 8-week HIIT training program (*p* = 0.002). Specifically, body fat exhibited a 1.21% reduction after the training intervention.

### 3.2. Physiological Adaptations and Performance Changes

The variables VO_2_max, [La^+2^]peak, HRpeak_,_ and PEs did not change significantly after the 8-week HIIT training compared with the baseline values (*p* > 0.05), while PSVO_2_max marginally failed to reach statistical significance (*p* = 0.062), (Table 3). Significant changes were found in PSVT_2_ (*p* = 0.05), as well as in both the 200 m (*p* < 0.001) and 1000 m (*p* = 0.01) performance test after the 8-week training period compared with the baseline values (Table 3).

### 3.3. Hormonal Responses

Standard curves of the ELISA analyses of all the factors examined had an R^2^ coefficient ranging between 0.97 and 1. Systemic responses of the various hormones examined after the HIIT program are shown in Table 4. Specifically, GH, IGF1, Cort, fT4, TSH and the ratios GH/IGF1, Testo/Cort, and fT4/TSH showed mild changes following HIIT training without reaching statistical significance (*p* > 0.05; Table 4). Interestingly, serum levels of Testo increased significantly after the 8-week training period compared with the baseline (pre) levels (*p* = 0.05; Table 4).

### 3.4. Molecular Adaptations

**Figure 2:**
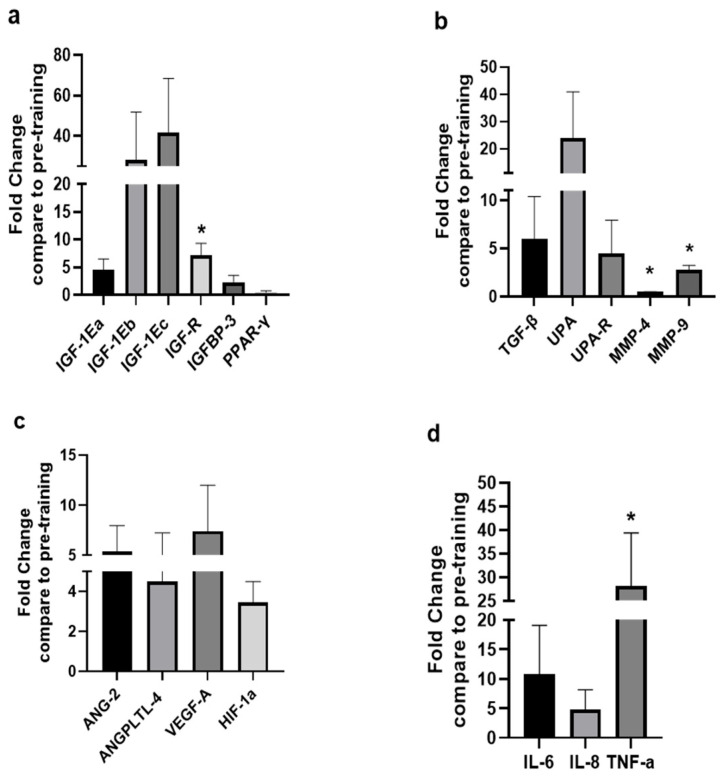
Expression levels of growth and remodeling, angiogenic, and inflammation-related factors after the HIIT training compared to pre-training. The diagrams represent the fold changes in the mRNA expression of (**a**) growth, (**b**) tissue remodeling, (**c**) angiogenic, and (**d**) inflammation-related factors in the trained (deltoid) skeletal muscles of the high-level kayak athletes following the 8-week high-intensity interval training (HIIT; see text for details). Data are presented as mean ± SD. Significantly different compared to pre-training levels: * *p* < 0.05. TGF-β: Transforming growth factor-beta; IGF-1Ea: Insulin-like growth factor 1Ea; IGF-1Eb: Insulin-like growth factor 1Eb; IGF-1Ec: Insulin-like growth factor 1Ec; IGF-1R: Insulin-like growth factor-1 receptor; IGFBP-3: Insulin-like growth factor binding protein-3; UPA: Urokinase type plasminogen activator; UPAR: Urokinase type plasminogen activator receptor; ANG-2: Angiopoietin 2; ANGPTL-4: Angiopoietin-like proteins; HIF-1a: Hypoxia inducible factor-1a; VEGF-A: Vascular endothelial growth factor-a; PPAR-γ: Peroxisome proliferator-activated receptor-γ; TNF-a: Tumor necrosis factor-a; IL-6: Interleukin 6; IL-8: Interleukin 8; MMP-4: Matrix metalloproteinase-4; MMP-9: Matrix metalloproteinases-9.

The changes in the expression levels of growth, angiogenic, and inflammation-related factors observed after the 8-week HIIT protocol are shown in Figure 2. Specifically, compared with the pre-exercise training values, the mRNA expression of the IGF-1 isoforms IGF-1Ea, IGF-1Eb, and IGF-1Ec, the IGF binding protein-3, and the type 1 IGF receptor (IGF-1R) increased, and that of PPAR-γ decreased (*p* = 0.076), though without reaching statistical significance (*p* > 0.05) due to a large inter-individual variability observed between the kayakists’ responses, except for the significant increase in the IGF-1R expression post-HII training (*p* < 0.05; Figure 2a). Similarly, after the completion of the HIIT exercise program the expression of TGF, UPA, UPA-R increased, again without these increases being statistically significant compared to pre-training values (*p* > 0.05), while significant changes were revealed in the expression levels of MMP-4 (decrease) and MMP-9 (increase) (*p* < 0.05; Figure 2b). Moreover, an overall increase was observed in the mRNA expression of the angiogenic factors examined, i.e., ANG-2, ANGPTL-4, VEGF-A, and HIF-1a (*p* = 0.074), but again these changes failed to reach significance due to the large individual variation in the expression responses regarding these factors after the post-HIIT exercise program (*p* > 0.05; Figure 2c). Moreover, an upregulation was revealed in the expression of the inflammation-related factors IL-6, IL-8, and TNF-a after the exercise training, with that of TNF-1 being significant (*p* < 0.05; Figure 2d).

## 4. Discussion

High-intensity interval exercise may represent a time-efficient strategy for eliciting physiological adaptations and performance improvements, and kayak athletes have leveraged these benefits [20,39]. The present study concurrently investigated changes in physiological and performance variables, alongside alterations in hormonal and gene expression levels of key growth, remodeling, angiogenic, and inflammation-related factors, following an 8-week HIIT protocol in national-level flatwater kayakists. Following the intervention, athletes exhibited measurable improvements in both performance and physiological markers: VO_2_max increased by 2.86%, PSVO_2_max on the kayak ergometer increased by approximately 4.28%, PSVT2improved by 4.52%. Concurrently, serum testosterone levels increased by 15.8%, while muscle gene expression analysis revealed upregulation of key anabolic and angiogenic markers, including IGF-1 and VEGF-A, alongside downregulation of pro-inflammatory genes such as IL-6 and TNF-α. These quantified results indicate that HIIT elicited directional anabolic, angiogenic, and anti-inflammatory adaptations, providing a clear basis for interpreting the molecular and performance responses observed in this cohort.

Specifically, our study showed that PSVT2, which represents the paddling speed at the point of anaerobic threshold (VT2), was significantly increased after the completion of the HIIT protocol. These findings corroborate that high-intensity training is an effective stimulus for the improvement of aerobic and anaerobic metabolism and VT2 in kayakists [20,40]. Notably, aerobic metabolism is highly activated in high-intensity interval exercise bouts, especially during the last efforts [2], suggesting that HIIT can be effective in inducing both cardiovascular and muscular adaptations [2,39]. Although very little is known about how the high-level kayak athletes respond to a HIIT program, it seems that HIIT can elicit significant improvements of endurance variables, such as PSVT2, in these athletes [40]. Indeed, HIIT models can greatly improve variables relating to aerobic capacity, such as VO_2_max and VT2 [20,40]; nevertheless, the physiological mechanisms through which HIIT leads to aerobic adaptations are yet not clearly characterized [39]. Moreover, and interestingly, in this study, body fat exhibited a significant reduction after the HIIT protocol, implying that it can be effective in increasing oxidative phosphorylation and fatty acid oxidation [1,2,8].

Although participants demonstrated clear performance improvements, including enhanced PSVT2 (peak speed at lactate threshold) and faster 200 m and 1000 m flatwater times, VO_2_max remained largely unchanged. This pattern suggests that the HIIT-induced adaptations were more peripheral and sport-specific rather than central [9,13]. In kayak sprint athletes, upper-body muscular strength, power, and neuromuscular efficiency are critical determinants of short- and middle-distance performance, meaning improvements in muscle contractile properties, capillary density, and anaerobic energy contribution can enhance performance without necessarily altering maximal oxygen uptake [25,41]. Additionally, increases in lactate threshold and enhanced oxidative capacity at submaximal intensities could allow athletes to sustain higher paddling speeds without a measurable increase in VO_2_max [20]. Thus, the dissociation between VO_2_max and performance outcomes highlights the importance of specific muscular and metabolic adaptations in explaining HIIT efficacy in upper-body-dominant sports like sprint kayaking [25,41].

Notably, aerobic metabolism is highly activated in high-intensity interval exercise bouts, especially during the last efforts [2], suggesting that HIIT can be effective in inducing both cardiovascular and muscular adaptations [39]. In particular, although very little is known about how the high-level kayak athletes respond to a HIIT program, it seems that HIIT can elicit significant improvements of endurance variables, such as PSVT2, in these athletes [39]. Indeed, HIIT models can greatly improve variables relating to aerobic capacity, such as VO_2_max and VT2 [40]; nevertheless, the physiological mechanisms through which HIIT leads to aerobic adaptations are not yet clearly characterized [20]. Moreover, and interestingly, in this study body fat exhibited a significant reduction after the HIIT protocol, implying that it can be effective in increasing oxidative phosphorylation and fatty acid oxidation [1,8].

Moreover, as HIIT activates both aerobic and anaerobic energy systems [2,20], we assumed that the improvement of PSVT2 could potentially contribute to the physiological mechanism(s) that mediate the improvement in performance tests of 1000 m and 200 m Olympic distance found in this study. It has been suggested that HIIT training could be a useful strategy to achieve greater improvements in certain variables of kayaking performance and the significant decrease in the time needed to complete the T1000 m and T200 m tests following the HIIT protocol potentially indicates a more effective training model to improve performance in these kayak Olympic distances.

Along with the changes in the kayaking performance variables, exercise is a powerful stimulus for the endocrine system, modifying the circulating levels of many hormones, including steroid hormones testosterone and cortisol [42,43,44,45,46]. Testosterone is a major anabolic hormone, while cortisol produces generally catabolic effects and manages the stress response. Thus, the testosterone/cortisol (T/C) ratio has been considered a good indicator of anabolic/catabolic exercise responses and adaptations [42]. The present study revealed no significant changes in the T/C ratio, though increased serum testosterone levels were observed following the 8-week HIIT protocol, indicating the potential involvement of this hormone in anabolic adaptive mechanisms to high-intensity interval training, although conflicting results have been reported for testosterone responses following HIIT [42].

In addition, only mild systemic responses of Cort, GH, IGF1, fT4, and TSH and no changes in the ratios GH/IGF1 and fT4/TSH were found following the HIIT protocol used in this study. However, it should be mentioned that the post-training blood as well as muscle biopsy samples were taken 48 h after the last exercise bout, in order to detect only the training effect and not the acute responses to HIIT exercise. An exercise bout represents physical stress and induces an acute disturbance of the body’s homeostasis, particularly in the exercised muscles but also in other organs, while in the recovery phase homeostasis is re-established [43,44,45]. Thus, the findings of this study suggest that, overall, acute endocrine responses rather than chronic adaptations might be expected following such an 8-week HIIT protocol.

Furthermore, the present study revealed moderate to significant changes in the expression of tissue growth and remodeling, and inflammation- and angiogenesis-related factors post-HIIT compared to the pre-training expression levels. High-intensity exercise-induced loading of skeletal muscle can stimulate the activation of tissue growth and the extracellular matrix (ECM) remodeling program [46] which includes growth, pro- and anti-inflammatory factors, and factors of the TGF-β/UPA/UPA-R system [47,48,49,50,51,52]. Skeletal muscle can undergo growth and remodeling following mechanical overloading, which is one of the proposed mechanisms by which exercise training leads to skeletal muscle hypertrophy [48,49]. More specifically, skeletal muscle growth and hypertrophy can be attributed to various physiological mechanisms, triggering anabolic drive for the muscle, which is accompanied by an increase in anabolic factors or depressing protein catabolic processes [49].

Exercise enhances anabolic pathways and local expression of IGF-1 promotes skeletal muscle growth [53,54]. Different IGF-1 mRNA isoforms, namely IGF-1Ea, IGF-1Eb, and IGF-1Ec, are produced by alternate splicing of the human IGF1 gene, while IGF-1 acts primarily through its receptor IGF-1R [53,54]. Interestingly, differential biologic activities have been reported for the different IGF-1 isoforms in the regulation of muscle regeneration and hypertrophy [53,54]. Biologic actions of IGF-1 are modulated by IGFBPs, while IGFBP-3 particularly provides tissue specificity for the local action of IGF-1 and it can regulate its local bioavailability in the tissues [54].

To the authors’ best knowledge, this is the first study investigating the mRNA expression responses of the main components of the IGF-1 bioregulation system after HIIT in skeletal muscle of high-level kayakists. Specifically, we found evidence that specific components responded differentially to HIIT, as these athletes presented disproportionally higher expression levels of the IGF-1Eb and IGF-1Ec isoforms compared to the more abundant isoforms IGF-1Ea and IGFBP-3, though without reaching statistical significance due to a high inter-individual variability observed among the subjects. However, HIIT influenced significantly the expression of IGF-1R. These findings might support the notion of IGF-1 isoform-specific actions during exercise-induced skeletal muscle adaptation [53,54].

In parallel with the effects of HIIT on the expression of the anabolic IGF-1 system components, we examined the potential effects of this type of exercise training on the induction of tissue remodeling, and angiogenesis- and inflammation-related factors as a part of the muscle adaptation process. Interestingly, tissue remodeling, and hypertrophic and angiogenetic pathways can be activated by local muscle inflammation, orchestrating muscle growth [47,48,49,50,51,52]. Indeed, muscle cells and a variety of other cells secrete cytokines, including IL-6, IL-8, and TNF-1α, remodeling factors, such as UPA, UPA-R, TGF-β, and MMPs, and angiogenic factors, as has been previously shown particularly following HIIT training [49] to potentially facilitate exercise-induced tissue remodeling, hypertrophy, and angiogenesis [50].

The present study showed an upregulation of pro-inflammatory factors TNF-α and IL-8, as well as IL-6, which acts as both a pro-inflammatory cytokine and an anti-inflammatory myokine, along with similar mRNA responses of the TGF-β/UPA/UPA-R tissue remodeling system, and alterations in the expression levels of MMP-9 and MMP-4 post-HIIT program [49]. These findings possibly imply a coregulation mechanism of these responses within the context of ECM remodeling that may occur after HIIT training. More specifically, TGF-β activation is facilitated by the binding of UPA to its receptor UPA-R. Moreover, the action of the UPA/plasmin system results also in the activation of MMPs, which are responsible for the extracellular degradation of proteins within the ECM, with MMP-9 playing a central role in ECM degradation in skeletal muscle and other tissue remodeling [49], as well as in angiogenesis and neovascularization [49,50]. Overall, these findings may indicate the activation of intramuscular inflammation and ECM remodeling programs following this type of exercise training in the elite kayakists.

More specifically, TGF-β activation is facilitated by the binding of UPA to its receptor UPA-R. Moreover, the action of the UPA/plasmin system results also in the activation of MMPs, which are responsible for the extracellular degradation of proteins within the ECM, with MMP-9 playing a central role in ECM degradation in skeletal muscle and other tissue remodeling [47,48,49,50,51,52] as well as in angiogenesis and neovascularization [50]. Overall, these findings may indicate the activation of intramuscular inflammation and ECM remodeling programs following this type of exercise training in the elite kayakists. An efficient remodeling of skeletal muscle requires a coordination between remodeling and inflammatory factors, and components of the TGF-β/UPA/UPA-R system have been reported to contribute to ECM degradation and reconstitution and, thus, to skeletal muscle remodeling [47,48,49,50,51,52].

Furthermore, the expression of several angiogenesis-related genes following the HIIT program was also investigated in this study. An intramuscular angiogenic program appeared to be triggered, as indicated by the upregulation of major pro-angiogenic factors, i.e., ANG-2, ANGPTL-4, HIF-1a, and VEGF-A [50]. However, the expression responses of these factors did not reach statistical significance as they exhibited a high variation among subjects, thus not permitting us to draw firm conclusions about their particular contribution to an angiogenic process post-HIIT program.

Overall, the representative molecular signature of the intramuscular cytokine, tissue remodeling, and angiogenic factors revealed in this study following HIIT may imply a functional relation of intramuscular inflammation to muscle remodeling and growth adaptations [47,48,49,50,51,52,53,54] following this type of exercise training in elite kayakists. However, more studies are required to further examine and reveal the potential role of inflammation, tissue remodeling, and angiogenesis programs following HIIT-induced muscle adaptations in these athletes.

The findings of this study have several practical implications for coaches and athletes in flatwater sprint kayaking. First, the demonstrated improvements in peak power, upper-body strength, and endurance parameters indicate that short, high-intensity interval sessions can efficiently enhance sport-specific performance, making HIIT a time-effective alternative to traditional volume-based training [13,20]. The concurrent increases in serum testosterone and upregulation of anabolic and angiogenic genes, along with reductions in inflammatory markers, suggest that HIIT not only improves performance but also supports favorable molecular adaptations that may enhance recovery and muscle remodeling [47,48,49,50,51,52,53,54]. Therefore, integrating 8-week HIIT protocols, such as 8 × 30 s efforts at ~120% VO_2_max with appropriate recovery periods, can be strategically applied during preparatory phases to maximize neuromuscular, cardiovascular, and metabolic adaptations while minimizing training volume [20]. Moreover, monitoring molecular and hormonal responses may help coaches individualize training intensity and recovery, optimizing both performance gains and athletes’ long-term health [1,3,13,20].

This study has some limitations; the small sample size in combination with the high inter-individual variability observed in transcriptional responses may have limited our potential to detect significant alterations in the expression of some of the multiple factors explored. Another limitation of the study is the absence of a control group. Each participant received the exercise training intervention, and its effects were compared with the pre-training “control” condition in each subject. Furthermore, the competitive level of the sprint kayak athletes—characterized by four years of training experience and national-level competition—restricts the generalizability of results to broader populations given their high baseline fitness. Moreover, the transcriptional nature of this study limits our ability to confirm the functional significance of the expression of these factors at the protein level and future studies are needed to evaluate these questions. Another limitation of the study is that the testing protocols took place in a simulator kayak ergometer, which could affect the validity of our findings. Moreover, in the present study, the measurements performed to determine potential adaptive responses to HIIT took place only once, 8 weeks after the training period and not at various time points during the training program, limiting the better characterization and thus the drawing of definite conclusions regarding the time-course(s) of the adaptations that occurred in functional, endocrine, growth and remodeling, angiogenic, and inflammation-related factors during the HIIT intervention period.

## 5. Conclusions

This study provided insights regarding the intramuscular molecular systemic, namely physiological and performance responses of key tissue remodeling and inflammatory factors to a HIIT training program in elite kayakists, contributing to the characterization of a potential network that regulates the various HIIT-induced responses in these athletes. The findings of the study demonstrated that the HIIT program resulted in improvement of physiological and performance variables in these athletes along with changes in serum testosterone and the skeletal muscle molecular signature of HIIT adaptations. Given that this type of exercise training has gained significant ground in kayak training, these findings may be a useful resource for future studies to further describe the systemic and skeletal muscle molecular signature of adaptive remodeling during and after different HIIT kayak training programs. Such a characterization could contribute to a better understanding of the adaptation mechanisms following HIIT, which may induce measurable long-term molecular and functional changes in kayak athletes. Larger, controlled trials are warranted for the development of training strategies targeting the improvement of various components of the biological processes that regulate HIIT-induced systemic and skeletal muscle adaptations.

## Figures and Tables

**Table 1:** The forward (F) and reverse (R) primer sequences presented in the table correspond to the genes selected for quantitative real-time PCR (qRT-PCR) analysis of skeletal muscle samples obtained from kayak athletes. The target genes included in the analysis were TGF-β (Transforming Growth Factor-β), IGF-1Ea/IGF-1Eb/IGF-1Ec (Insulin-Like Growth Factor-1 isoforms Ea, Eb, Ec), IGF-1R (Insulin-Like Growth Factor-1 Receptor), IGFBP-3 (Insulin-Like Growth Factor Binding Protein-3), VEGF-A (Vascular Endothelial Growth Factor-A), ANG-2 (Angiopoietin-2), ANGPTL-4 (Angiopoietin-Like Protein 4), HIF-1α (Hypoxia-Inducible Factor-1 alpha), TNF-α (Tumor Necrosis Factor-α), IL-6 (Interleukin-6), IL-8 (Interleukin-8), MMP-4 (Matrix Metalloproteinase-4), MMP-9 (Matrix Metalloproteinase-9), and PPAR-γ (Peroxisome Proliferator-Activated Receptor-γ). The housekeeping gene GAPDH (Glyceraldehyde-3-Phosphate Dehydrogenase) was used as an internal control for normalization of expression levels.

Target Gene	Primer Sequence
TGF-β	F: 5′-CTACTACGCCAAGGAGGTCAC-3′ R: 5′-ATGGAGTCGTTGGCCACGA-3′
VEGF-A	F: 5′-AGGGCAGAATCATCACGAAG-3′ R: 5′-CACACAGGATGGCTTGAAGA-3′
IGF-1 Ea	F: 5′-GTGGAGACAGGGGCTTTTATTTC-3′ R: 5′-CTTGTTTCCTGCACTCCCTCTACT-3′
IGF-1 Eb	F: 5′-ATGTCCTCCTCGCATCTCT-3′ R: 5′-CCTCCT TCTGTTCCCCTC-3′
IGF-1 Ec	F: 5′-CGAAGTCTCAGAGAAGGAAAGG-3′ R: 5′-ACAGGTAACTCGTGCAGAGC-3′
UPA	F: 5′-GTCTACCTGGGTCGCTCAAG-3′ R: 5′-CAGTGGTGGTTTTACGACAC-3′
UPA-R	F: 5′-CATGCAGTGTAAGACCAACGGGGA-3′ R: 5′-TGAGACCGGCCCGACAGTGGTAT-3′
IGF-1R	F: 5′-GGGAATGGAGTGCTGTATG-3′ R: 5′-CACAGAAGCTTTCGTTGAGAA-3′
IGFBP-3	F: 5′-AGTGAGTCGGAGGAAGACCGCA-3′ R: 5′-TCTCCCAGGCTACACCACCAAGG-3′
ANG-2	F: 5′-GACGGCTGTGATGATAGAAATAGG-3′ R: 5′-GACTGTAGTTGGATGATGTGCTTG-3′
ANGPTL-4	F: 5′-GTGGCTCAAACACCTGACCA-3′ R: 5′-GAAAGGGGGCTTCTCCAGTC-3′
HIF-1	F: 5′-AAACTTGGCAACCTTGGATTGG-3′ R: 5′-TCCGTCCCTCAACCTCTCAG-3′
PPAR-γ	F: 5′-TGCTCAAGTATGGTGTCCATGAG R: 5′-AGTGCATTGAACTTCACAGCAAA
TNF-a	F: 5′-AAGAGTTCCCCAGGGACCTCT-3′ R: 5′-ACATGGAGTAGATGAGGGT-3′
IL-6	F: 5′-CCTGACCCAACCACAAATGC-3′ R: 5′-ATCTGAGGTGCCCATGCTAC-3′
IL-8	F: 5′-CCACCGGAAGGAACCATCTC-3′ R: 5′-TTCCTTGGGGTCCAGACAGA-3′
MMP-4	F: 5′-TTTGGACACATCTGGGCAGT-3′ R: 5′-GGGCAGCCATAGAAGGTGT-3′
MMP-9	F: 5′-CAGGGAATGAGTACTGGGTCTATT-3′ R: 5′-ACTCCAGTTAAAGGCAGCATCTAC-3′
GAPDH	F: 5′-CATCACTGCCACCCAGAAGA-3′ R: 5′-TCCACCACCCTGTTGCTGTA-3′

**Table 2:** Somatometric characteristics of the kayak athletes before (Pre) and after (Post) completion of the high-intensity interval training (HIIT) program. The variables measured included body fat percentage (%BF), body mass index (BMI), height, total body mass, and lean body mass (LBM). Data are expressed as mean ± standard deviation (SD), *p*-values from paired *t*-tests and Cohen’s d size effects indicate the statistical significance of changes between Pre and Post measurements.

Variable	HIITpre	HIITpost	*t*-Test	Cohen’s d	Effect Size
Body Fat (%)	11.80 ± 2.94	10.59 ± 3.08	0.002 **	−0.39	Small–medium ↓
BMI (kg·m^−2^)	22.78 ± 2.45	22.48 ± 2.36	0.83	−0.12	Negligible
Height (cm)	176.33 ± 4	176.33 ± 4	–	0.00	No change
Body Mass (kg)	70.75 ± 7.13	69.81 ± 6.81	–	−0.13	Negligible
LBM (kg)	62.26 ± 4.80	62.26 ± 4.83	0.90	0.00	No change

Significantly different compared with pre values: ** *p* < 0.01. BMI: Body Mass Index, %BF: body fat percentage, LBM: lean body mass.

**Table 3:** Physiological and performance variables measured before (Pre) and after (Post) the high-intensity interval training (HIIT) program in kayak athletes. The variables included maximal oxygen uptake (VO_2_max), peak speed at VO_2_max (PSVO_2_max), speed at the second ventilatory threshold (PSVT_2_), peak blood lactate concentration ([La^+^]), peak exercise speed (PEs), peak heart rate (HRpeak), and 200 m and 1000 m time-trial performances. Data are expressed as mean ± standard deviation (SD); *p*-values from paired *t*-tests and Cohen’s d size effects are reported to show differences between Pre and Post testing.

Variables	HIITpre	HIITpost	*t*-Test (*p*)	Cohen’s d	Effect Size
VO_2_max (mL·kg^−1^·min^−1^)	46.96 ± 5.45	47.85 ± 3.37	0.593	+0.19	Small ↑
PSVO_2_max (km·h^−1^)	12.86 ± 1.23	13.38 ± 0.80	0.062	+0.49	Small–medium
PSVT2 (km·h^−1^)	11.10 ± 0.53	11.60 ± 0.10	0.050 *	+1.16	Large
[La^+2^]peak (mmol·L^−1^)	13.80 ± 2.78	14.74 ± 3.14	0.823	+0.32	Small
PES (–)	9.27 ± 0.80	9.47 ± 0.55	0.116	+0.29	Small
HR_peak_ (beats·min^−1^)	193.0 ± 10.17	193.0 ± 10.20	0.771	0.00	No change
T 1000 m(s)	247.53 ± 2.68	243.35 ± 2.10	0.0027 **	−1.71	Large
T 200 m(s)	39.76 ± 0.21	39.00 ± 0.21	0.0004 ***	−3.62	Very large

Significantly different compared with pre values: * *p* ≤ 0.05, ** *p* < 0.01, *** *p* < 0.001. VO_2_max: maximum oxygen uptake; PSVO_2_max: paddling speed at maximum oxygen uptake; PSVT2: paddling speed at ventilatory threshold 2; [La^+2^]peak: lactate accumulation 3 min after the VO_2_max test; PEs: paddling economy speed (speed at 75% of VO_2_max); HRpeak: heart rate at VO_2_max; T 1000 m: time needed to cover 1000 m Olympic distance; T 200 m: time needed to cover 200 m Olympic distance.

**Table 4:** Systemic concentrations of selected anabolic, catabolic, and thyroid-related hormones measured before (Pre) and after (Post) the high-intensity interval training (HIIT) intervention in kayak athletes. The variables included growth hormone (GH), insulin-like growth factor-1 (IGF-1), testosterone (Testo), cortisol (Cort), free thyroxine (fT4), and thyroid-stimulating hormone (TSH), as well as their respective ratios (GH/IGF-1, Testo/Cort, and fT4/TSH). Data are expressed as mean ± standard deviation (SD); *p*-values from paired *t*-tests and Cohen-d size effects are provided to indicate pre-post differences.

Variable	HIITPre	HIITpost	*t*-Test	Cohen’s d	Effect Size
GH (ng·μL^−1^)	1663.68 ± 1455.65	771.65 ± 1148.36	0.241	−0.69	Medium–large **↓**
IGF-1 (pg·μL^−1^)	312.27 ± 68.71	313.92 ± 56.17	0.934	+0.03	Negligible
GH/IGF-1 ratio	5.18 ± 4.74	2.53 ± 3.91	0.250	−0.61	Medium
Testosterone (ng·μL^−1^)	4.86 ± 0.43	5.63 ± 0.87	0.050 *	+1.08	Large
Cortisol (ng·μL^−1^)	89.73 ± 55.43	88.53 ± 12.52	0.964	−0.03	Negligible
Testo/Cort ratio	0.054 ± 0.029	0.064 ± 0.008	0.869	+0.47	Small–medium
fT4 (nmol·μL^−1^)	84.38 ± 11.31	85.22 ± 10.25	0.779	+0.08	Negligible
TSH (μIU·μL^−1^)	1.87 ± 0.20	2.07 ± 0.40	0.470	+0.61	Medium
fT4/TSH ratio	45.1 ± 21.18	41.18 ± 24.85	0.741	−0.17	Small

Significantly different compared with the pre values: * *p* ≤ 0.05. GH: growth hormone; IGF-1: insulin-like growth factor 1; Testo: testosterone; Cort: cortisol; TSH: thyroid-stimulating hormone, and fT4: free tetraiodothyronine (thyroxine).

## Data Availability

The data presented in this study are available on request from the corresponding author.

## Abbreviations

The following abbreviations are used in this manuscript:

Testo	Testosterone
fT4	Free Tetraiodothyronine (Thyroxine)
HIIT	High-intensity interval training
TSH	Thyroid Stimulating Hormone
Cort	Cortisol
GH	Growth Hormone
BF	Body fat
BMI	Body mass index
BM	Body mass
LBM	lean body mass
FM	Fat mass
HRpeak	Peak heart rate
VO_2_max	Maximal oxygen consumption
VT2	Ventilatory threshold 2
PSVO_2_max	Paddling speed at maximal oxygen uptake
PES	Paddling economy speed
PSVT2	Paddling speed at ventilatory threshold 2
T 1000 m	time needed to cover 1000 m Olympic distance
T 200 m	time needed to cover 200 m Olympic distance
TGF-β	Transforming growth factor-beta
VEGF-A	Vascular endothelial growth factor-a
IGF-1Ea	Insulin-like growth factor 1Ea
IGF-1Eb	Insulin-like growth factor 1Eb
IGF-1Ec	Insulin-like growth factor 1Ec
IGF-1R	Insulin-like growth factor-1 receptor
IGFBP-3	Insulin-like growth factor binding protein-3
UPA	Urokinase type plasminogen activator
UPA-R	Urokinase type plasminogen activator receptor
ANG-2	Angiopoietin 2
ANGPTL-4	Angiopoietin-like proteins
HIF-1a	Hypoxia inducible factor-1a
PPAR-γ	Peroxisome proliferator-activated receptor-γ
TNF-a	Tumor necrosis factor-a
IL-6	Interleukin 6
IL-8	Interleukin 8
MMP-4	Matrix metalloproteinase-4
MMP-9	Matrix metalloproteinases-9
GAPDH	Glyceraldehyde 3-phosphate dehydrogenase
